# Dissection of Pilus Tip Assembly by the FimD Usher Monomer

**DOI:** 10.1016/j.jmb.2012.12.024

**Published:** 2013-03-11

**Authors:** William J. Allen, Gilles Phan, Scott J. Hultgren, Gabriel Waksman

**Affiliations:** 1 Institute of Structural and Molecular Biology, University College London and Birkbeck College, Malet Street, London WC1E 7HX, UK; 2 Center for Women's Infectious Disease Research, Washington University School of Medicine, 660 South Euclid Avenue, Campus Box 8230, St. Louis, MO 63110, USA

**Keywords:** Nte, N-terminal extension, DSE, donor strand exchange, NTD, N-terminal domain, CTD, C-terminal domain, AUC, analytical ultracentrifugation, DDM, *n*-dodecyl-β-d-maltoside, uropathogenic *E. coli*, chaperone, membrane transporters, kinetics, fimbriae

## Abstract

Type 1 pili are representative of a class of bacterial surface structures assembled by the conserved chaperone/usher pathway and used by uropathogenic *Escherichia coli* to attach to bladder cells during infection. The outer membrane assembly platform—the usher—is critical for the formation of pili, catalysing the polymerisation of pilus subunits and enabling the secretion of the nascent pilus. Despite extensive structural characterisation of the usher, a number of questions about its mechanism remain, notably its oligomerisation state, and how it orchestrates the ordered assembly of pilus subunits. We demonstrate here that the FimD usher is able to catalyse *in vitro* pilus assembly effectively in its monomeric form. Furthermore, by establishing the kinetics of usher-catalysed reactions between various pilus subunits, we establish a complete kinetic model of tip fibrillum assembly, able to account for the order of subunits in native type 1 pili.

## Graphical Abstract

**Figure:**
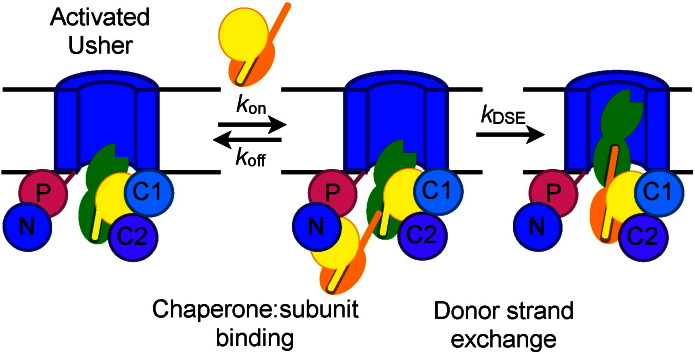

**Highlights:**

► The mechanism of pilus tip assembly by FimD is investigated. ► The FimD usher can catalyse donor strand exchange in its monomeric form. ► A novel model for the kinetics of donor strand exchange is proposed. ► A complete kinetic characterisation of pilus biogenesis is achieved.

## Introduction

The ability to recognise and attach to host cells is a key step in the infection process of many pathogenic bacteria. In Gram-negative bacteria, this initial infection event is often carried out by specialised surface-exposed fibres, termed “pili”, which are anchored in the bacterial outer membrane and extend out from the cell surface. Specific adhesin proteins at the distal tips of these pili bind tightly to targets on the surface of host cells and allow colonisation to take place. The most common pathway for the biogenesis of adhesive pili is the chaperone–usher pathway. In this system, individual pilus subunits are secreted into the bacterial periplasm by the general secretory pathway and then folded by specific periplasmic chaperones and carried to the outer membrane, where they are assembled into pili by an outer membrane assembly platform known as the usher.^1,2^

One of the best-studied examples of the chaperone–usher pathway is the Fim system of uropathogenic *Escherichia coli*, which produces type 1 fimbriae that mediate attachment to human bladder epithelial cells.^3,4^ Type 1 pili are made up of four distinct subunit types (Fig. S1a): the narrow tip fibrillum comprises a single copy each of the adhesin FimH and the two adaptor subunits FimG and FimF—in that order—followed by several thousand copies of the major pilus subunit FimA, wound together helically to form the pilus rod.

The structure of each individual pilus subunit is an incomplete immunoglobulin-like fold where the last β-strand, strand G, is missing, leaving a deep hydrophobic groove across the protein surface.^5,6^ Within the periplasm, prior to pilus assembly, this groove is occupied by the G1 strand of the chaperone FimC,^7,8^ in an interaction known as donor strand complementation (Fig. S1b). Upon pilus assembly, the G1 strand of FimC is replaced by an N-terminal extension (Nte) of 10–20 residues from the next subunit in assembly, a mechanism termed donor strand exchange^9–11^ (DSE; Fig. S1c). DSE is energetically favourable, thanks to the relative instability of the chaperone:subunit complex and the extreme stability of the groove:Nte interaction between adjacent subunits.^12^

Pilus assembly *in vivo* is catalysed by the usher, FimD,^13,14^ a multi-domain outer membrane protein comprising a 24-stranded β-barrel pore,^15–17^ a soluble periplasmic N-terminal domain (NTD) with high affinity for chaperone:subunit complexes,^18,19^ two periplasmic C-terminal domains (CTD1 and CTD2), which are necessary for pilus biogenesis,^20–22^ and which have recently been shown to form an additional binding site for chaperone:subunit complexes,^17^ and a central plug domain, which blocks the pore when it is not in use (Fig. S1d). Pilus formation is initiated by binding of the FimC:FimH chaperone:subunit complex to the usher NTD, followed by displacement of the plug into the periplasm and concomitant insertion of the FimH lectin domain into the pore. Subsequent chaperone:subunit complexes—first FimC:FimG, then FimC:FimF, and finally multiple copies of FimC:FimA—are then recruited to the usher NTD where they undergo DSE with the previously assembled subunit. As the pilus forms, it is threaded through the β-barrel domain of the usher and then assumes its final quaternary structure.^16,23^

The exact mechanism through which the usher catalyses DSE is not yet fully understood; however, two plausible models have been suggested based on recent structural data.^16,17^ The first was put forward to explain structural and biochemical evidence that the usher functions as a dimer:^16,24,25^ in this model (Fig. S1e), only one pore is used for secretion^16^ but two NTDs are required for chaperone:subunit complex recruitment. At any given time, one of the two NTDs is bound to the chaperone:subunit complex at the base of the pilus, so a second is required to recruit the chaperone:subunit complex next in assembly.

Recently, however, a crystal structure of FimD:FimC:FimH with all usher domains present has revealed a second chaperone:subunit binding site on the C-terminal domains and suggested an alternative mechanism for pilus biogenesis that only requires a single usher molecule^17^ (Fig. S1f). In this model, chaperone:subunit complexes are initially recruited to the usher NTD, where they undergo DSE before being passed over to the CTDs—thus freeing the NTD to recruit the next chaperone:subunit complex.

In the study presented here, we investigate the oligomeric state of the apo FimD usher and of FimD bound to FimC:FimH using analytical ultracentrifugation (AUC) and show that while apo FimD is mostly dimeric over the range of concentration studied, FimD bound to FimC:FimH is mostly monomeric, even at high concentration. We then investigate the kinetics of usher-mediated incorporation of FimG or FimF at a concentration of FimD:FimC:FimH where FimD is monomeric and demonstrate that FimD is an effective catalyst of DSE in its monomeric form. We next investigate the concentration dependence of the DSE reaction, leading to the characterisation of a previously unknown conformational state. Finally, we compare the rates of cognate and non-cognate DSE reactions catalysed by the usher and show that usher catalysis is sufficient to account for the specificity of subunit ordering observed in native pili. Thus, a complete kinetic characterisation of the subunit incorporation cycle during pilus biogenesis by the Fim system is presented, an unprecedented result for any membrane-embedded nanodevice.

## Results

### Monomeric *versus* dimeric state of the FimD usher in the FimD:FimC:FimH complex

**Fig. 1:**
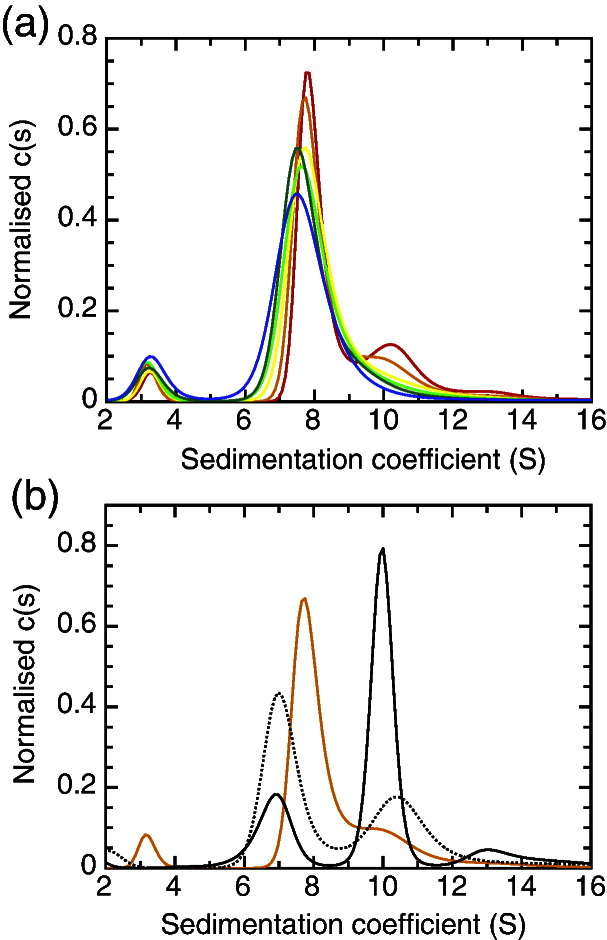
AUC of usher complexes. (a) *c*(*s*) profiles at six different FimD:FimC:FimH concentrations, normalised by integral. The concentrations are 4.28 μM (red), 2.57 μM (orange), 1.37 μM (yellow), 0.86 μM (green), 0.51 μM (teal), and 0.17 μM (blue). (b) Normalised *c*(*s*) profiles of FimD alone, both at high (5 μM; black continuous line) and at low (2 μM; black dotted line) concentrations. For comparison, the 2.57-μM FimD:FimC:FimH trace from (a) is also shown (orange).

In order to determine the oligomerisation state of the purified FimD:FimC:FimH complex, we carried out AUC sedimentation velocity experiments at a variety of different FimD:FimC:FimH concentrations. At the highest concentration tested (4.3 μM), the main population sedimented at *s* = 7.99 S (*s*_20,w_ = 8.18 S), equivalent to a molecular mass of 163 kDa if the best-fit frictional ratio of 1.34 is used and the contribution from detergent is ignored (red trace in Fig. 1a). This corresponds closely to the mass of a FimD:FimC:FimH complex where FimD is monomeric (145 kDa) and is substantially less than the predicted mass of a FimD_2_:FimC:FimH complex where FimD is dimeric (238 kDa). As bound detergent would lead us to overestimate the total mass of protein in the complex (see SI.1 for details), we are confident that the large peak at *s* = 7.99 S corresponds to the FimD:FimC:FimH complex with the monomeric and not the dimeric usher.

At the higher protein concentrations (> 1 μM), a second population is also visible centred at *s* = 10.47 S, equivalent to 244 kDa and corresponding to the dimeric usher complex FimD_2_:FimC:FimH, although we cannot rule out FimD_2_ based on these data. Nevertheless, FimD_2_ appears to be the minority form in solution when FimC:FimH is present, even at our highest concentration (4.3 μM). The maximum concentration of protein we could test was limited by the absorbance optics of the AUC; however, by integrating the monomer and dimer peaks at 4.3 μM FimD:FimC:FimH, we estimate the *K*_d_ of usher dimerisation to be of the order of 20 μM.

In addition to the populations arising from the usher, we also see a small FimC:FimH peak at *s* = 3.20 S, corresponding to a molecular mass of 41.3 kDa. This compares reasonably with the calculated mass 52.6 kDa and suggests some rapid reversible dissociation of the FimD:FimC:FimH complex.

### Apo FimD oligomerisation state

In order to test whether the presence of FimC:FimH was having any effect on the dimerisation of the usher, we ran sedimentation velocity experiments on FimD alone—that is, in the absence of chaperone:adhesin complex. The resulting *c*(*s*) distributions at 5 μM (continuous line) and 2 μM (dotted line) are shown in Fig. 1b. Whereas FimD in complex with FimC:FimH is almost exclusively monomeric over the entire concentration range tested (up to 4.3 μM), FimD alone shows a significant proportion of dimers at both concentrations tested and the dimeric form is more abundant at high concentration. Although we hesitate to calculate a dimerisation constant from these results—apo FimD tends to aggregate over time, and so the exact monomer:dimer ratio depends on the age of the sample at the time of loading—it is nonetheless clear that the presence of FimC:FimH reduces the propensity of FimD to dimerise.

### Kinetics of catalysed DSE by a monomeric usher

**Fig. 2:**
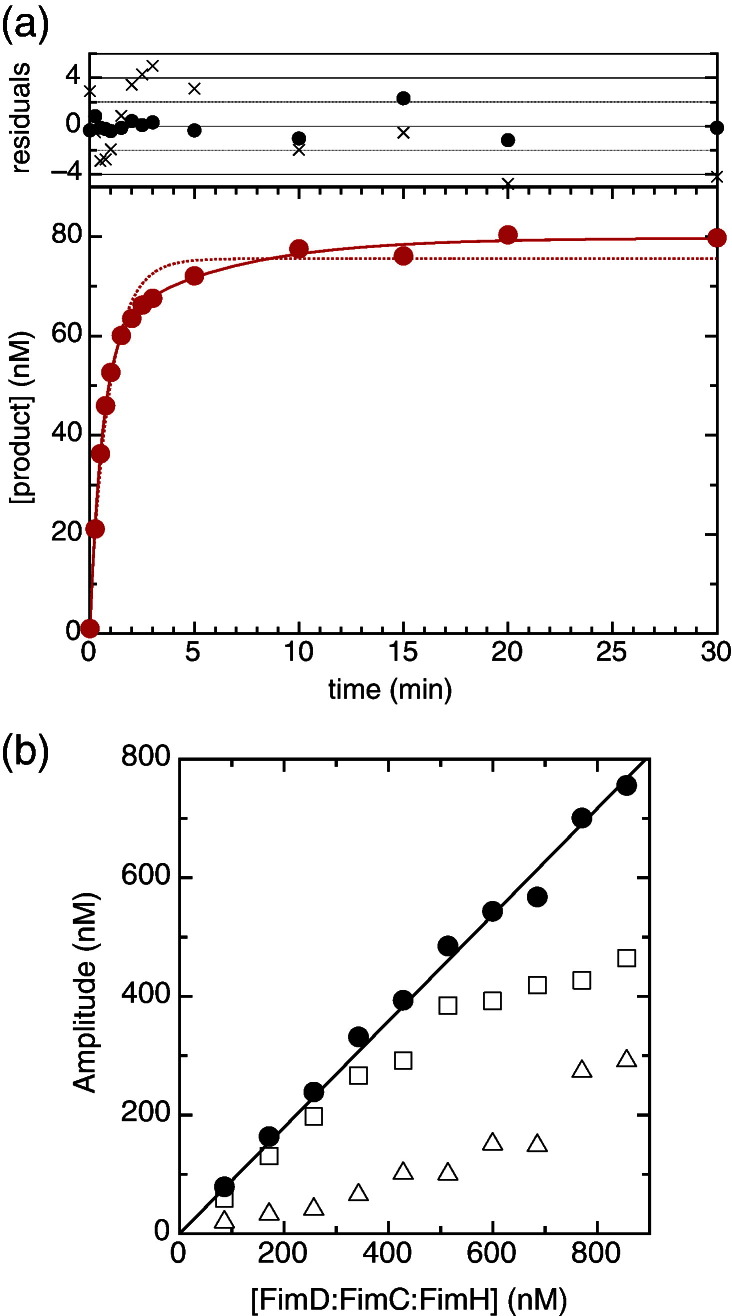
Kinetics of DSE with FimC:FimG at 4 °C. (a) DSE carried out at 4 °C using 85.6 nM FimD:FimC:FimH complex and 1 μM labelled FimC:FimG^S92C[A647]^. The formation of the FimH:FimG^S92C[A647]^ product is plotted as a function of reaction time and fitted both to a single exponential (dotted line; crosses in the residual plot) and to a double exponential (continuous line; circles in the residual plot). (b) Amplitudes of the fast (*A*_1_; open squares) and slow (*A*_2_; open triangles) exponentials as a function of FimD:FimC:FimH concentration. The sum of the two amplitudes is shown as black filled circles.

Having investigated the concentration range in which FimD in the FimD:FimC:FimH complex is monomeric (below ~ 1 μM), we used a previously described DSE assay to ask whether a monomeric usher is active in catalysing DSE.^17^ Figure 2a shows a time course for the formation of the stable FimH:FimG product following the rapid mixing of 85.6 nM FimD:FimC:FimH and 1 μM labelled FimC:FimG (FimC:FimG^S92C[A647]^) at 4 °C. From the amount of FimH:FimG product formed, we can conclude that 90% of the FimD:FimC:FimH complexes are active in incorporating FimG. Importantly, DSE takes place even at FimD:FimC:FimH concentrations at which the usher is monomeric. This effectively demonstrates that a monomeric usher is fully competent to catalyse DSE. It also rules out dimerisation as the origin of the biphasic kinetics discussed below.

As can be seen directly and from the residual plot in Fig. 2a, the data fit poorly to a single exponential (dotted line), but well to a double exponential (continuous line), implying at least one additional step beyond substrate binding and DSE (see SI.2 for details). Many possible reaction pathways can give rise to biphasic kinetics. To distinguish between them, we performed a series of DSE assays at different FimD:FimC:FimH concentrations (but within a range where FimD is primarily monomeric; see above) while keeping the FimC:FimG concentration constant but always in excess (Fig. S2a). Note that because we are running a gel with fluorescently labelled FimG, we elected to vary the concentration of the non-fluorescent component, that is, FimD:FimC:FimH. The rates of the two exponential phases are essentially concentration independent (Fig. S2b); this is unsurprising as we have an excess of FimC:FimG and are varying FimD:FimC:FimH; that is, we are working at or close to pseudo-first-order kinetics. Interestingly, however, the amplitudes show a non-linear dependence on FimD:FimC:FimH concentration (Fig. 2b). The sum of the two amplitudes (black circles) is linearly related to the concentration of FimD:FimC:FimH—confirming that the product formed is proportional to the amount of the limiting complex provided—however, the individual amplitudes are not linearly related to FimD:FimC:FimH concentration; rather, *A*_1_ (squares in Fig. 2b) dominates at lower substrate concentrations, while *A*_2_ (triangles in Fig. 2b) becomes more significant as the concentration of FimD:FimC:FimH increases. This additional step must occur before or in parallel with substrate binding; otherwise, we would not see an effect of concentration on the relative amplitudes. As a secondary, very slow binding mode seems structurally and mechanistically implausible, we interpret this amplitude effect as a slow equilibrium step before substrate binding, that is, where two slowly interchanging forms of FimD:FimC:FimH complex exist, only one of which is able to bind substrate.

The simplest model for *in vitro* DSE that both fits our data and is consistent with prior knowledge can therefore be described by the following model equation:

(1) $$\mathrm{DCHx}\overset{k_{+1}}{\underset{k_{-1}}{\Leftrightarrow }}\mathrm{DCH}+\mathrm{CG}\overset{k_{\mathrm{on}}}{\underset{k_{\mathrm{off}}}{\Leftrightarrow }}\mathrm{DCH}\cdot \mathrm{CG}\overset{k_{\mathrm{DSE}}}{\Rightarrow }\mathrm{DCHG}+C$$

This model contains five different rate constants: *k*_+_ _1_ and *k*_−_ _1_ describe the slow pre-equilibrium required to explain the amplitude data (with *DCHx* unable to bind substrate); *k*_on_ and *k*_off_ represent binding and dissociation of FimC:FimG to the active FimD:FimC:FimH population, presumably to the NTD of FimD; and *k*_DSE_ is the rate of DSE itself. In order to confirm the validity of this model and extract individual rates, we modelled the reaction using KinTek Explorer (KinTek). The use of simulation software allows us to fit the data directly to a reaction scheme without the simplifications required to extract rates numerically. By fixing the rates *k*_on_ = 0.34 nM^−^ ^1^ min^−^ ^1^ and *k*_off_ = 11,800 min^−^ ^1^ (as determined previously at 4 °C^26^), we were able to determine the remaining three rates as *k*_+_ _1_ = 0.197 ± 0.017 min^−^ ^1^, *k*_−_ _1_ = 0.0355 ± 0.0053 min^−^ ^1^, and *k*_DSE_ = 65.8 ± 4.7 min^−^ ^1^ at 4 °C. These values represent the averages and standard deviations from fitting the 10 different data sets shown in Fig. S2a. The values obtained do not show any systematic dependence on FimD:FimC:FimH concentration, further validating our choice of model. The affinities determined in Ref. 26 were confirmed in our laboratory using surface plasmon resonance (see SI.3) and shown to be unaffected by the presence of *n*-dodecyl-β-d-maltoside (DDM).

### Rates of FimF incorporation by the FimD:FimC:FimH:FimG complex

FimF is the next subunit after FimG to be incorporated into the nascent type 1 pilus (Fig. S1a). We therefore measured the rate of DSE for FimC:FimF by FimD:FimC:FimH:FimG. To do so, we produced a fluorescently labelled FimF (FimF^Q99C[A647]^) and carried out DSE assays between unlabelled FimD:FimC:FimH:FimG complex and labelled FimC:FimF^Q99C[A674]^. Position Q99 on FimF was chosen as it is the equivalent of S92 in FimG (as determined by structural comparison using DaliLite). At the concentrations and temperature (4 °C) used for FimC:FimG incorporation, we saw very little product (FimG:FimF^Q99C[A647]^) formation over the course of 30 min (data not shown). However, by increasing the reaction temperature to 20 °C, and using 500 nM usher complex with 5 μM chaperone:subunit complex, we were able to follow the reaction to completion (Fig. S4a). As in the case of FimG incorporation, the data fit better to a double than to a single exponential, although the difference in fit is less pronounced (Fig. S4a). Because the reaction with FimC:FimF is so slow compared to incorporation of FimG into FimD:FimC:FimH, we performed two additional control assays: (i) using unlabelled proteins and silver staining to monitor FimH:FimG:FimF product formation (Figs. S3b and S4b) and (ii) using FimD:FimC:FimH:FimG^S92C[A647]^ and unlabelled FimC:FimF (Fig. S4b). All three experiments yield very similar results, indicating that DSE is not inhibited by the presence of a label on incoming FimF or the already incorporated FimG.

Repetition of the FimD:FimC:FimH:FimG *versus* FimC:FimF^Q99C[A647]^ assay at four different FimC:FimF concentrations (Fig. S4c) reveals the same dependence of amplitude on concentration as for FimG incorporation by the FimD:FimC:FimH complex, suggesting a similar conformational state prior to chaperone:subunit binding. These data were thus fitted to Eq. (1), again using KinTek Explorer, but this time using the on- and off-rates for FimC:FimF at 20 °C^26^ (*k* = 1.08 nM^−^ ^1^ min^−^ ^1^ and *k*_off_ = 8520 min^−^ ^1^). From the average of the four data sets, we calculate *k*_DSE_ to be 3.16 ± 0.25 min^−^ ^1^ with *k*_+_ _1_ = 0.139 ± 0.032 min^−^ ^1^ and *k*_−_ _1_ = 0.036 ± 0.024 min^−^ ^1^. To corroborate this rate further, we carried out a series of DSE assays using labelled FimD:FimC:FimH:FimG^S92C[A647]^ and unlabelled FimC:FimF, which yielded similar results (Fig. S4d).

**Fig. 3:**
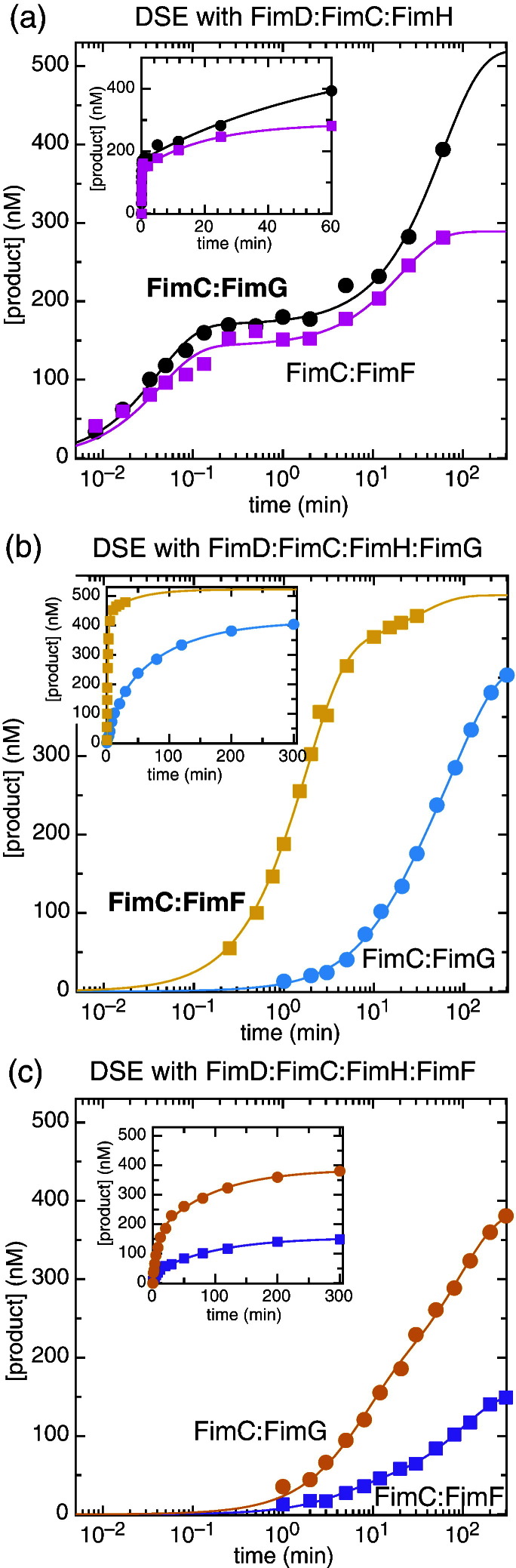
Kinetics of cognate and non-cognate DSE reactions at 20 °C. All reactions are performed at 20 °C with 500 nM usher complex and 5 μM chaperone:subunit complex, fitted to double exponentials (continuous lines), and shown with the *x*-axis in logarithmic time (with insets showing real time). (a) Incorporation of FimF^Q99C[A647]^ (non-cognate subunit; magenta squares) compared with correct (cognate) FimG^S92C[A647]^ incorporation (black circles) by FimD:FimC:FimH. (b) Incorporation of FimG (non-cognate) by FimD:FimC:FimH:FimG^S92C[A647]^ (cyan circles). For comparison, correct (cognate) incorporation of FimF^Q99C[A647]^ by FimD:FimC:FimH:FimG is also shown (yellow squares). Note that labelling of FimG in the FimD:FimC:FimH:FimG^S92C[A647]^ does not alter the rate of the reaction (Fig S4b). (c) Incorporation of FimG (non-cognate) by FimD:FimC:FimH:FimF^Q99C[A647]^ (orange squares) and of FimF^Q99C[A647]^ (also non-cognate) by FimD:FimC:FimH:FimF (purple squares).

Reaction rates determined at different temperatures cannot be compared: therefore, in order to compare FimC:FimG and FimC:FimF incorporation directly, we measured the rate of FimC:FimG incorporation under the same conditions as used for FimC:FimF, using a rapid-quench apparatus (KinTek). The results of the reaction of 5 μM FimC:FimG^S92C[A647]^ with 500 nM FimD:FimC:FimH at 20 °C are shown in Fig. 3a (black circles). In this case, no residual plots are required to see that two exponentials are a better fit than one: the first phase is extremely fast (*k*_obs1_ = 23.2 min^−^ ^1^), while the second phase is 3 orders of magnitude slower (*k* = 0.0164 min^−^ ^1^).

The data set for FimC:FimG at 20 °C was fitted to the model in Eq. (1) with KinTek Explorer, using the values *k*_on_ = 1.71 nM^−^ ^1^ min^−^ ^1^ and *k*_off_ = 51,300 min.

Although these values have not been determined directly, they were chosen as they correspond to the known *K*_d_ at 20 °C (30 μM^26^) and are 5 times faster than the rates at 4 °C, in keeping with the temperature dependence of binding of the other chaperone:subunit complexes.^26^ Fitting in this manner gives a value of 2.85 ± 0.78 s^−^ ^1^ for *k*_DSE_, equivalent to 171 min, 55 times faster than equivalent incorporation of FimF opposite FimG.

### Rates of non-cognate DSE reactions

In order to understand the basis of subunit ordering in the mature pilus, it is not sufficient to know the rates of the cognate DSE reactions (by “cognate”, we mean the reactions that are predicted to occur from our knowledge of subunit ordering in the mature pilus, i.e., FimG into FimH, and FimF into FimG); the rates of non-cognate DSE must also be determined. To this end, we carried out DSE reactions between the following: (i) FimD:FimC:FimH and FimC:FimF^Q99C[A647]^ (incorporation of FimF into FimH; magenta squares in Fig. 3a; quench flow was used for this experiment as it proceeds too quickly to quench manually), (ii) FimD:FimC:FimH:FimG^S92C[A647]^ and FimC:FimG (incorporation of FimG into FimG; cyan circles in Fig. 3b), (iii) FimD:FimC:FimH:FimF^Q99C[A647]^ and FimC:FimG (incorporation of FimG into FimF; orange circles in Fig. 3c), and (iv) FimD:FimC:FimH:FimF and FimC:FimF^Q99C[A647]^ (incorporation of FimF into FimF; purple squares in Fig. 3c). We used FimD:FimC:FimH:FimG^S92C[A647]^ or FimD:FimC:FimH:FimF^Q99C[A647]^ and unlabelled FimC:FimG for reactions (ii) and (iii) because, these reactions being slow, spontaneous formation (non-usher mediated) of FimG polymers during the course of the experiment is observed. However, it should be noted that a label on the penultimate subunit does not affect DSE (Fig. S4b). As with the cognate reactions, we found best fits to double exponential kinetics and were able to extract rates from Eq. (1) using KinTek Explorer. Note that while the activity of FimD:FimC:FimH:FimF is lower than that of the other usher complexes, its reaction kinetics are still readily determined. The complete set of *k*_DSE_ results is summarised in Table 1.

## Discussion

Until recently, the usher was thought to function primarily as a dimer, and several lines of biochemical and structural evidence suggest that it does indeed form dimers within the outer membrane.^16,24,25^ However, the crystal structure of the usher FimD in complex with the chaperone:adhesin complex FimC:FimH suggested a plausible mechanism for pilus assembly that does not require a second usher molecule to be present^17^ (see mechanism in Fig. S1f). Here, we have measured the dimerisation of detergent-solubilised FimD:FimC:FimH complex directly, using AUC, and show that it is primarily monomeric at concentrations below 5 μM. However, our DSE assay, performed in the same detergent, shows that the usher is able to assemble pili at FimD:FimC:FimH concentrations of 500 nM and lower. Thus, we conclude that dimerisation is not required for pilus assembly once FimC:FimH is bound. Note that we did not investigate the kinetics of FimD_2_:FimC:FimH complex where the usher is dimeric because concentrations where the FimD_2_:FimC:FimH species is predominant (well over 20 μM according to our AUC experiments) cannot be obtained (the complex aggregates). Intriguingly, however, we show that FimD alone has a higher propensity to dimerise in the absence of FimC:FimH than in its presence; we speculate that this effect may be relevant to the energetics or regulation of the initiation step under physiological conditions.

In order to fully understand the role the usher plays in pilus assembly, the rates of catalysed DSE for each different pilus subunit are required,^26^ along with their affinities for the usher NTD and their relative concentrations in the periplasm. Using our recently published single-turnover DSE assay,^17^ and fitting to the simplest reaction model able to describe the data [Eq. (1)], we have now determined *k*_DSE_ for all six possible DSE reactions involving the tip fibrillum components FimH, FimG, and FimF. As the affinities of the matching chaperone:subunit complexes are already known^26^ (and unchanged in the presence of detergents), as is the steady-state rate of FimD-catalysed FimA polymerisation,^15^ these results represent the final pieces in the jigsaw of usher catalysis. We find that, at 20 °C, insertion of FimG into the FimH subunit binding groove is very fast (*k*_DSE_ = 171 min^−^ ^1^), whereas insertion of FimF into the FimG subunit binding groove is 55 times slower at 3.16 min^−^ ^1^. Surprisingly, we also find that non-cognate insertion of FimF opposite FimH (58.3 min^−^ ^1^) is only 3 times slower than the cognate insertion of FimG.

In addition to the rates at which DSE occurs from the substrate-bound complex (*k*_DSE_), we have also identified two states of the usher prior to substrate binding. These states appear to be present in all *in vitro* catalysed DSE reactions, albeit with different parameters depending on the temperature and subunit pair. It is not clear from our data what structural event might give rise to this pre-equilibrium: it cannot be FimD dimerisation because the dimer is essentially unpopulated at the concentration used. Nor do we believe it to be binding and dissociation of FimC:FimH: DSE assays carried out in the presence of additional FimC:FimH do not show significantly altered kinetics to those with a 1:1:1 FimD:FimC:FimH ratio (unpublished observations). Thus, it is likely to be either a slow structural rearrangement intrinsic to FimD itself, blocking substrate binding, or some form of substrate inhibition—that is, tight binding of chaperone subunit complexes in a non-productive manner. Note that a FimD form unable to bind chaperone:subunit complexes correctly would effectively be a paused state, its presence increasing the total time it takes to form the pilus but having no effect on the relative rates of the various steps.

An important question is the extent to which the usher specifically catalyses cognate interactions: does it recognise correct subunit pairs and preferentially match them together, or does it merely amplify innate properties of the different Nte sequences and subunit binding groove structures? Our results presented here show that with usher catalysis, the cognate reaction of FimG into FimH is 3-fold faster than the non-cognate FimF into FimH reaction, while the cognate FimF into FimG reaction is 13-fold faster than the non-cognate FimG into FimG reaction. Differences in the uncatalysed rates of DSE for different subunits and Ntes have been investigated extensively using the Pap system, and it has been shown that each Nte binding groove reacts with its cognate Nte(s) between 2 and 50 times faster than with any other non-cognate Nte.^27^ Interestingly, therefore, although DSE rates are hundreds of times faster when catalysed by the usher,^15^ the differences between correct and incorrect DSE are remarkably similar whether the reaction is catalysed or not. We thus conclude that the intrinsic structural properties of the pilus subunits coupled with the sequences of the Ntes are the main determinants of *k*_DSE_ for usher-catalysed DSE.

**Fig. 4:**
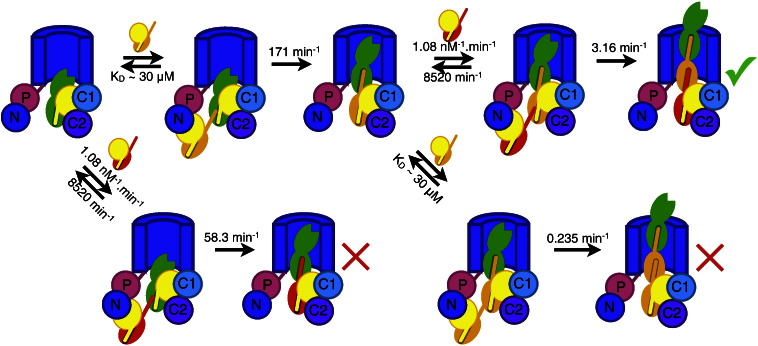
A complete model for the formation of the tip fibrillum at 20 °C—given the three components FimD:FimC:FimH, FimC:FimG, and FimC:FimF—is shown with all reaction rates. The usher barrel and NTD are shown in blue, with the plug, CTD1, and CTD2 in pink, cyan, and purple, respectively. FimC is in yellow, FimH is in green, FimG is in orange, and FimF is in red. The on- and off-rates for FimC:FimF, as well as the *K*_d_ of FimC:FimG, are taken from the literature,^26^ while the rates of DSE were determined in this work. The correct reaction pathway is shown along the top, with binding and incorporation of FimC:FimG followed by FimC:FimF. Possible misincorporation reactions (incorporation of FimC:FimF by FimD:FimC:FimH or incorporation of FimC:FimG by FimD:FimC:FimH:FimG) are also shown.

By combining the kinetics results obtained here with the known affinities of the chaperone:subunit complexes for the usher NTD, we can now present a complete model of formation of the tip fibrillum with all the relevant rates (Fig. 4; Table 1). The model in Fig. 4 contains both the on-pathway reactions (DSE of FimG into FimH and of FimF into FimG) and the possible off-pathway reactions (DSE of FimF into FimH and of FimG into FimG). By simulating this complete model using KinTek Explorer, we can determine the amount of correct *versus* incorrect product formed at completion for any given set of substrate concentrations. Intriguingly, because of the low selectivity of the FimH binding groove for FimG over FimF and the higher affinity of the usher NTD for FimF, equimolar concentrations of FimG and FimF yield significantly more incorrect FimH:FimF pilus tips than correct FimH:FimG:FimF tips (Fig. S5a). Only by reducing the concentration of FimC:FimF by several times do we produce levels of correct tip approaching those observed *in vivo* (Fig. S5b). Indeed, our simulation with a 10-fold excess of FimC:FimG over FimC:FimF provides results very compatible with the data of Hahn *et al.*, who observed FimG or FimF in 80% of tip fibrillums and both FimF and FimG in 70%.^4^ Our results thus support the hypothesis that the intrinsic rates of DSE between the subunit binding grooves and Ntes, together with the affinities of the chaperone:subunit complexes for the usher NTD and their respective expression levels in the periplasm, are sufficient to account for subunit ordering in the type 1 pilus. However, they also suggest that the order of subunits in the Fim pilus tip is less strictly controlled than previously suspected.

DSE of the major pilus subunit, FimA, with FimH, FimG, FimF, or itself has been investigated previously.^15^ FimA undergoes DSE 10-fold faster with FimF than it does with FimH or FimG. This result is consistent with the known order of pilus subunit that positions FimA after FimF. However, DSE of FimA with FimF is itself 10-fold slower than the slowest DSE rate observed with tip fibrillum subunits: clearly, once the tip fibrillum is assembled, commitment to the assembly of the FimA rod is rate limiting. However, once the first FimA is reacted with FimF, incorporation of subsequent FimA subunits is extremely fast, over 4-fold faster than the incorporation of FimG into FimH, the fastest rate in tip fibrillum subunit assembly.

The study presented here therefore concludes the complete kinetic description of usher-mediated pilus biogenesis. Uniquely for any membrane transporter, the usher can be extracted from the membrane, purified to homogeneity, and be made to work *in vitro*,^15,17^ thereby allowing the collection of a unique set of kinetic data leading to the complete characterisation of the subunit incorporation cycle, the first for a membrane-embedded nanodevice. No doubt such results will prove instrumental in understanding other membrane transporters and will also be used in the design of compounds specifically targeted to block crucial steps during pilus biogenesis.

## Materials and Methods

### Protein expression, purification, and labelling

FimD, FimD:FimC:FimH, FimC:FimG, and FimC:FimF were expressed and purified as described previously.^17^ FimC:FimG with a fluorescent Alexa 647 label at position 92 (FimG^S92C[A647]^) has also been described previously.^17^ A plasmid containing FimC:FimF^Q99C^ was designed using standard techniques (see Supplementary Methods for details), and FimC:FimF^Q99C[A647]^ was purified and labelled in the same manner as FimC:FimG^S92C[A647]^. FimD:FimC:FimH:FimG and FimD:FimC:FimH:FimF (both labelled and unlabelled) were produced by mixing the FimD:FimC:FimH complex with FimC:FimG, FimC:FimG^S92C[A647]^, FimC:FimF, or FimC:FimF^Q99C[A647]^ at a 1:1.5 ratio and then gel filtrating (Superose-12 column, GE Healthcare) in a buffer consisting of 50 mM Tris, pH 8.0, 150 mM NaCl, 1 mM ethylenediaminetetraacetic acid, and 0.05% DDM (Fig. S2a).

### Analytical ultracentrifugation

AUC was carried out using a Beckman Coulter ProteomeLab XL-I ultracentrifuge. Samples were exchanged by gel filtration (Superose-12 column) into a buffer consisting of 50 mM Tris, pH 8.0, 150 mM NaCl, 1 mM ethylenediaminetetraacetic acid, and 0.05% DDM, and then immediately transferred to sample cells for an An50Ti rotor. Gel-filtration buffer was used as a reference in each case. Sedimentation velocity measurements were performed overnight at 20 °C, at a speed of 42,000 rpm, using absorbance optics. Data were fitted globally to a continuous *c*(*s*) distribution as implemented by SedFit,^28^ using the following parameters: *v̅* (specific volume of the protein) = 0.7249 cm^3^ g^−^ ^1^, as calculated from the amino acid sequence using SEDNTERP; ρ (buffer density) = 1.00718 g cm^−^ ^3^, measured directly using an Anton Paar DMA 5000 density meter; and μ (buffer viscosity) = 0.01002 P, calculated for buffer without DDM using SEDNTERP (the contribution of DDM to μ is negligible at the concentration used^29^).

### DSE assays

The basic DSE assay has been described previously.^17^ DSE between subunits results in highly stable complexes that are resistant to denaturation by SDS, provided that they are not boiled. Our DSE assay exploits this by mixing purified FimD:chaperone:subunit or FimD:chaperone:subunit:subunit complexes with purified chaperone:subunit complexes (either cognate or non-cognate) at time 0, quenching the DSE reaction with HCl at various time points, and then monitoring formation of the subunit:subunit or subunit:subunit:subunit products by SDS-PAGE electrophoresis.

As one of the subunits is fluorescently labelled, the DSE product band can easily be quantified as described in Phan *et al.*^17^ For the rapid-quench DSE assay, used for the fast reactions at 20 °C, this protocol was modified to make use of a quench flow apparatus (KinTek). Solutions of FimD:FimC:FimH complex and FimC:FimG^S92C[A647]^ or FimC:FimF^Q99C[A647]^ were prepared at 2 × the final concentration, mixed rapidly at a 1:1 ratio, and then quenched with 500 mM HCl. Quenched samples were flash frozen in liquid nitrogen prior to analysis by gel electrophoresis and fluorescence imaging, in order to limit any potential time-dependent artefacts. Flash freezing was also used for normal DSE reactions with time courses longer than 30 min.

The DSE assay with FimD:FimC:FimH:FimG and FimC:FimF (both unlabelled) was carried out as with labelled protein and then analysed by densitometry (see Supplementary Methods for details).

## Figures and Tables

**Table 1:** Rates of DSE for all subunit pairs

		Acceptor subunit			
		FimH	FimG	FimF	FimA
Donor subunit	FimG	**171 ± 47**	0.235 ± 0.084	0.326 ± 0.161	n.d.
	FimF	58.3 ± 15.7	**3.16 ± 0.25**	0.359 ± 0.059	n.d.
	FimA	0.0024^⁎^	0.0048^⁎^	**0.03^⁎^**	960*

All values are for *k*_DSE_ in min^−^ ^1^. Those values determined in this work are all at 20 °C, while those taken from Ref. 26 (indicated with *) are at 23 °C. Cognate interactions are denoted in boldface.
