# Supplementary material for: Dissection of Pilus Tip Assembly by the FimD Usher Monomer
Source: J Mol Biol. 2013 Mar 11;425(5):958–67. doi: 10.1016/j.jmb.2012.12.024 (PMC3650583; doi:10.1016/j.jmb.2012.12.024)
Supplement: Supplementary file 1 — Supplementary materials [file mmc1.pdf]

Supplementary Information

SI1. Effects of bound detergent on apparent protein molecular weight

The molecular weight of a protein can be calculated from sedimentation velocity data using the Svedberg equation:

(eq. S1) $$\frac{s}{D} = \frac{M(1-\bar{v}\rho)}{RT},$$

where s and D are the sedimentation coefficient and diffusion constant, respectively, extracted from the experimental data; R is the gas constant; T is the temperature; M is the molecular weight of the protein; $\bar{v}$ is the partial specific volume of the protein; and ρ is the buffer density. The presence of detergent bound to the protein will affect its apparent molecular weight such that:

(eq. S2) $$M_{tot} = M_p + M_d,$$

where M_{tot}, M_p and M_d signify the total molecular weight of the complex and those of protein and detergent, respectively. The partial specific volume of the complex ($\bar{v}_{tot}$) will be a weighted average of the protein $\bar{v}_p$ and the detergent $\bar{v}_d$:

(eq. S3) $$\bar{v}_{tot} = \bar{v}_p \frac{M_p}{M_{tot}} + \bar{v}_d \frac{M_d}{M_{tot}}.$$

By rearranging equation 1, and substituting in equations 2 and 3, we find that:

(eq. S4) $$\frac{sRT}{D} = M_p(1-\bar{v}_p\rho) + M_d(1-\bar{v}_d\rho).$$

In all our experiments, we have calculated an apparent molecular mass (M_{app}), using $\bar{v}_p$:

(eq. S5) $$\frac{sRT}{D} = M_{app}(1-\bar{v}_p\rho).$$

Combining equations 4 and 5, then rearranging, we can therefore show how M_{app} will be affected by differing amounts of detergent bound to the protein:

(eq. S6) $$M_{app} = M_p + M_d \frac{1-\bar{v}_d \rho}{1-\bar{v}_p \rho}$$

Given that $\bar{v}_p$ = 0.7249 $cm^3.g^{-1}$, $\bar{v}_d$ = 0.814 $cm^3.g^{-1}$ and ρ = 1.00718 $g.cm^{-3}$, we can calculate the exact impact of any given quantity of detergent bound to the protein:

(eq. S7) $$M_{app} = M_p + 0.67 M_d.$$

Thus, the presence of any detergent will lead us to overestimate the mass of protein by 0.67 times the mass of detergent, and any numbers we derive for the mass of complexes can be considered overestimates.

SI2. Derivation of expected kinetics of DSE

According to classical kinetics (see e.g. Fersht, 1985[1] for details) product formation for the general reaction:

$$DCH + CG \underset{k_{-1}}{\overset{k_{+1}}{\Leftrightarrow}} DCH \bullet CG \underset{k_{-2}}{\overset{k_{+2}}{\Leftrightarrow}} DCHG + C$$

should have a double exponential form. When k_{+1} and k_{-1} are fast compared to k_{+2} and k_{-2}, as is known to be the case for the donor strand exchange reaction, and when FimC:FimG is in large excess over FimD:FimC:FimH, these two exponentials have the rates:

(eq. S8) $$\lambda_1 = k_{-1} + k_{+1}[CG]$$

and

(eq. S9) $$\lambda_2 = k_{-2} + \frac{k_{+2}[CG]}{[CG]+K_D},$$

where $K_D = \frac{k_{-1}}{k_{+1}}$ and $[CG]$ is the concentration of FimC:FimG added.

Because k_{+1} and k_{-1} are known to be 5.7 x 10^6 $M^{-1}.s^{-1}$ and 197 s^{-1}, respectively at 4 °C[2], we can evaluate λ_1 directly. Regardless of FimC:FimG concentration this phase will have reached equilibrium within the dead time of mixing (~1 s), and will be unmeasurable over our timescale of experiment (15 s – 30 min). Thus, formation of product will take place with a single rate equal to $\frac{k_{+2}[CG]}{[CG]+K_D}$. Note that k_{-2} = 0, because donor strand exchange is irreversible. The amplitude of this phase will be equal to the total amount of product formed at completion, i.e. the concentration of FimD:FimC:FimH provided. As the observed rate of product formation follows double exponential kinetics (see main text), but a simple binding followed by DSE model only predicts a single exponential, an additional reaction step is required to account for the data.

SI3. Surface plasmon resonance measurements

In order to confirm that the affinity of the NTD of FimD for chaperone:subunit complexes is not affected by the detergent used to keep the entire usher soluble, we carried out SPR experiments using a Biacore 3000 instrument (GE healthcare). Through a capture coupling method[3], FimC:FimG or FimC:FimF complexes were covalently immobilised on a NTA chip via the hexahistidine tagged FimC. FimD-NTD was then flowed over the chip surface with a concentration range of 0.176 μM to 180 μM, in TBS-E buffer (20 mM Tris-HCl pH 8.0, 150 mM NaCl, 1 mM EDTA), ± DDM 0.05% (w/v). The flow rate was set at 5 μl/min with a contact time of 5 minutes and a dissociation time of 10 minutes. The affinity constants (K_D) were measured directly by the equilibrium binding analysis, at 20 °C. The equilibrium signals of the sensorgrams were plotted against the different concentrations of FimD-

NTD and the Langmuir binding equation with a 1 to 1 stoichiometry was fitted in order to determine the K_D. Data were evaluated using the software BIAeval (Biacore AB).

The SPR results are shown in Table S1. As can be seen, the presence of DDM at 0.05 % has a negligible effect on the affinity of the FimD NTD for either chaperone:subunit complex measured. Furthermore, our results are consistent with those determined previously by the Glockshüber group[2]. We are therefore confident that our use for data fitting of the numbers in ref 2 – determined at multiple temperatures and using multiple techniques – is appropriate.

DDM, 0.05% (w/v)	-	+
K_D, FimC:FimG (μM)	15	11
K_D, FimC:FimF (μM)	7	6

Table S1. Estimated affinities of the FimD NTD for chaperone:subunit complexes in the absence or presence of 0.05 % DDM, as determined by surface plasmon resonance (see SI3. for details)

Supplementary Methods

Production of FimC:FimF$^{Q99C[A647]}$

The mutation FimF^{Q99C} was introduced into FimF by site-directed mutagenesis using the QuikChange protocol (Stratagene), with the primers 5'-GGA ATA CAG CTT CTG AAT GAG TGC CAA AAT CAA ATA CCC CTT AAT GC-3' and 5'-GC ATT AAG GGG TAT TTG ATT TTG GCA CTC ATT CAG AAG CTG TAT TCC-3'. A plasmid expressing FimC:FimF^{Q99C} was produced by cloning FimC into the

plasmid vector pASK-IBA2 (IBA) using the restriction free cloning protocol[4] with the primers 5'-GGT TTC GCT ACC GTA GCG CAG GCC GGA GTG GCC TTA GGT GCG ACT CGC-3' and 5'-CTC GAA TTC GGG ACC ATG GTC TCC TTA ATG GTG ATG GTG ATG GTG TTC-3', then amplifying FimF^{Q99C} with the primers 5'-AGC TGA GGA TCC GCT GAA TGT CGT TAA GGG-3' and 5'-CCG ATA GGA TCC TTA CTG ATA TTC AAG AGT GAA GG-3' and inserting the gene into the BamH1 restriction site of the FimC-containing vector using standard molecular biology procedures. Correct cloning was confirmed by sequencing prior to expression in BL21 Star (DE3) cells (Invitrogen). FimC:FimF$^{Q99C[A647]}$ was produced in the same manner as FimC:FimG$^{S92C[A647]}$ (ref 5), with the exception that 100 μg.ml^{-1} ampicillin was used to maintain the plasmid and induction was carried out using 200 μg.ml^{-1} anhydrotetracycline.

Donor strand exchange with unlabelled protein

The donor strand exchange assay with FimD:FimC:FimH:FimG and FimC:FimF (both unlabelled) was carried out as with labelled protein, but protein bands were visualised by silver staining (SilverQuest, Invitrogen). Stained gels were then scanned and loaded into ImageGauge 4.0 (Fujifilm) for analysis. To quantify substrate and product bands, an intensity profile was measured for each lane (average intensity as a function of lane position), background estimated and subtracted, then the integrals for the regions corresponding to the bands calculated. These were converted into product formation by:

$$P = \frac{S_0}{(1-c)}\left(\frac{I_p}{I_s + I_p} - c\right),$$

where P is the concentration of product formed, S_0 is the initial concentration of the limiting substrate (FimD:FimC:FimH:FimG), I_p and I_s are the intensities of the product and substrate bands, respectively, and c is a correction factor equal to $\frac{I_p}{I_s + I_p}$ for FimD:FimC:FimH:FimG complex alone (i.e. with no FimC:FimF added). This correction factor was required to account for the fact that a small band is present at the position of product in the usher complex samples (corresponding to FimD:FimC:FimH:$FimG_2$ and arising from the protein production). The above analysis corrects for differences in intensity between the lanes and for small quantities of contaminants at the position of the product band. However it should be noted that due to difficulty in determining background and the assumption that stain intensity is proportional to amount of protein, this method is less accurate and quantitative than fluorescence.

Supplementary Figures

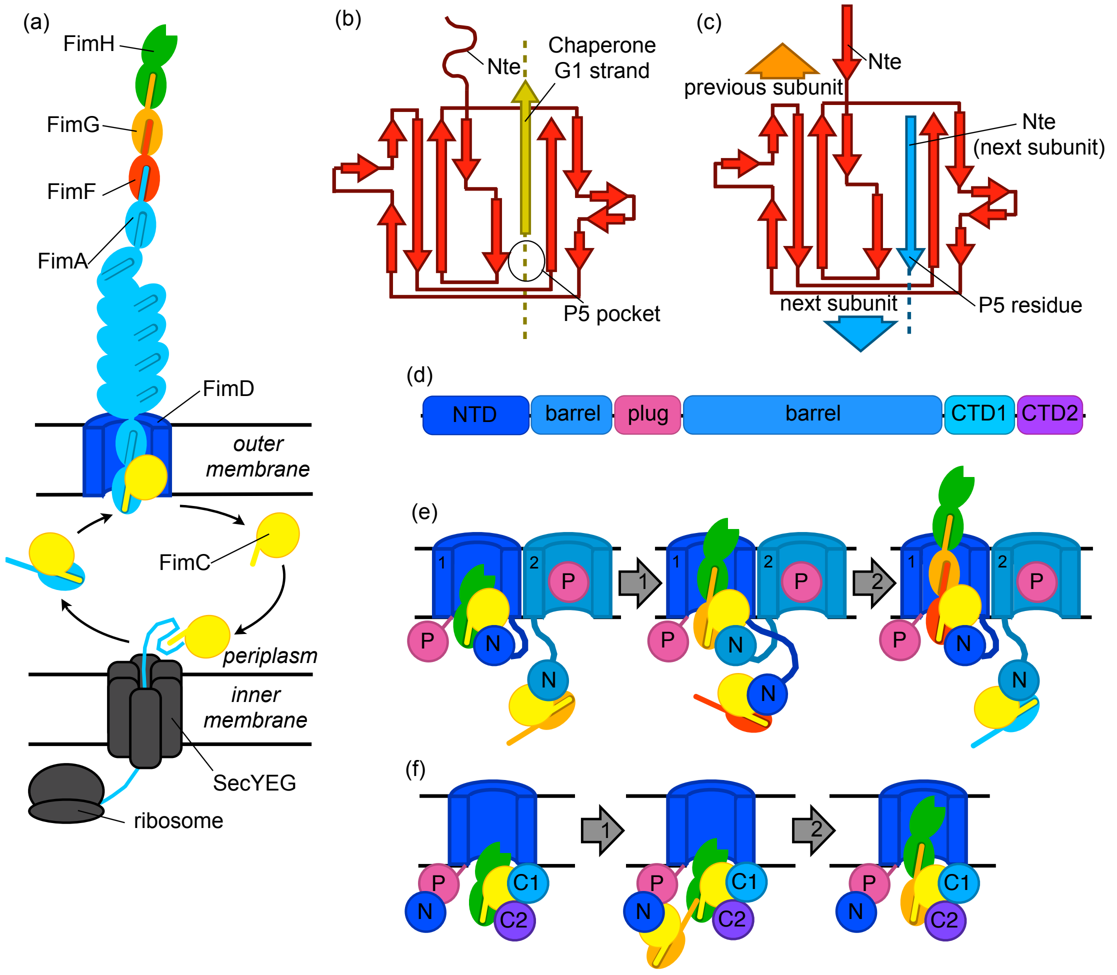

Fig. S1. The chaperone usher pathway. (a) Schematic diagram of a type 1 pilus and its assembly machinery. Pilus subunits are coloured red, orange, green and cyan for FimF, FimG, FimH and FimA respectively. The usher FimD is shown in blue, and the chaperone FimC is shown in yellow. See text for discussion. (b) Donor strand complementation. The G1 strand of the chaperone (yellow) supplies the missing strand in the C-terminally truncated Ig fold of the subunit (red). (c) Donor strand exchange. The N-terminal extension (Nte) of the next subunit in assembly (blue) supplies the missing strand of the subunit previously assembled (red). (d) Domain organization of FimD. (e) The dimer model of pilus assembly, as proposed by Remaut *et al*., 2008 (see text for details). The colour scheme is the same as panel (a), with the

addition of the NTD (blue; labelled N) and the plug (magenta; labelled P). The second usher is shown in lighter blue. The C-terminal domains (CTDs) are not represented because no density was observed for them and also their role was unknown. (f) The monomer model of pilus assembly, proposed by Phan *et al,* 2011[5] (see text for details). The colour scheme is the same as above, with the addition of CTD1 (cyan; labelled C1) and CTD2 (purple; labelled C2). The structure of FimD:FimC:FimH by Phan *et al*, 2011 revealed a second chaperone:subunit binding site formed by CTD1 and CTD2, in addition to the NTD. With two binding sites per usher molecule, pilus biogenesis can be executed by just one usher molecule.

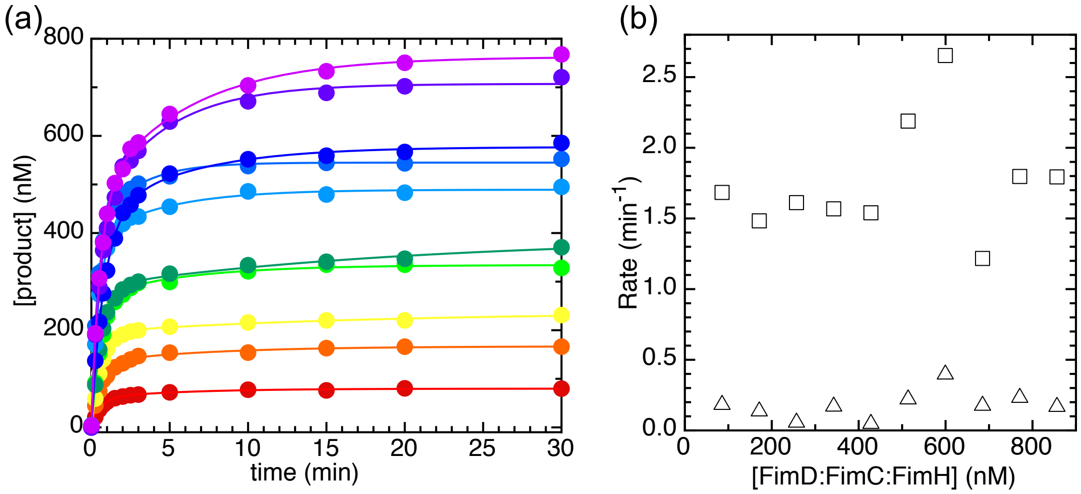

Fig. S2 (a) DSE assays as in Fig. 2A but with varying initial concentrations of FimD:FimC:FimH complex while keeping an excess of FimC:FimG$^{S92C[A647]}$. The concentrations of FimD:FimC:FimH are: 85.6 nM (red), 171 nM (orange), 257 nM (yellow), 342 nM (light green), 428 nM (dark green), 514 nM (cyan), 599 nM (blue), 685 nM (dark blue), 771 nM (purple) and 856 nM (violet). At all ten concentrations tested, data fit well to a double exponential (solid lines) and poorly to a single exponential. (b) Secondary plots derived from the results in panel A showing the rates of the fast (k_{obs1}; open squares) and slow (k_{obs2}; open triangles) phases as a function of FimD:FimC:FimH concentration.

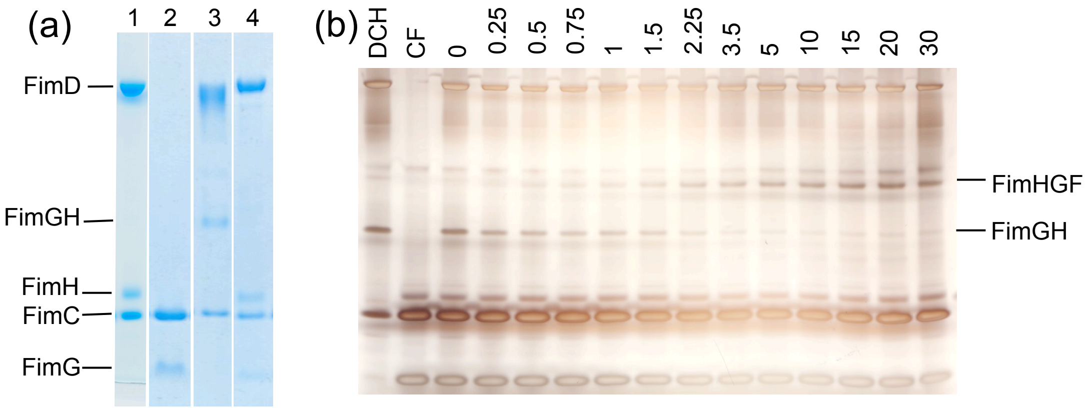

Fig. S3. Gels of donor strand exchange (a) A coomassie-stained SDS gel showing the production of the FimH:FimG product. The lanes are: (1) purified FimD:FimC:FimH complex; (2) purified FimC:FimG complex; (3) the product (FimH:FimG) of mixing (1) and (2) followed by gel filtration to remove excess FimC:FimG (unboiled); and (4) the same complex as in lane (3), but boiled prior to loading onto the gel. Because the DSE reaction product is stable in SDS but breaks apart in SDS followed by boiling, bands that disappear upon boiling are characteristic of DSE products. (b) A silver-stained SDS gel showing a DSE assay between 500 nM FimD:FimC:FimH:FimG complex and 5 μM FimC:FimF complex, both unlabelled. The lanes contain (from left to right) FimD:FimC:FimH:FimG only, FimC:FimF only, and the two mixed after various time points (time shown in minutes). The depletion of the FimH:FimG band and the formation of a new band corresponding to FimH:FimG:FimF is clearly visible.

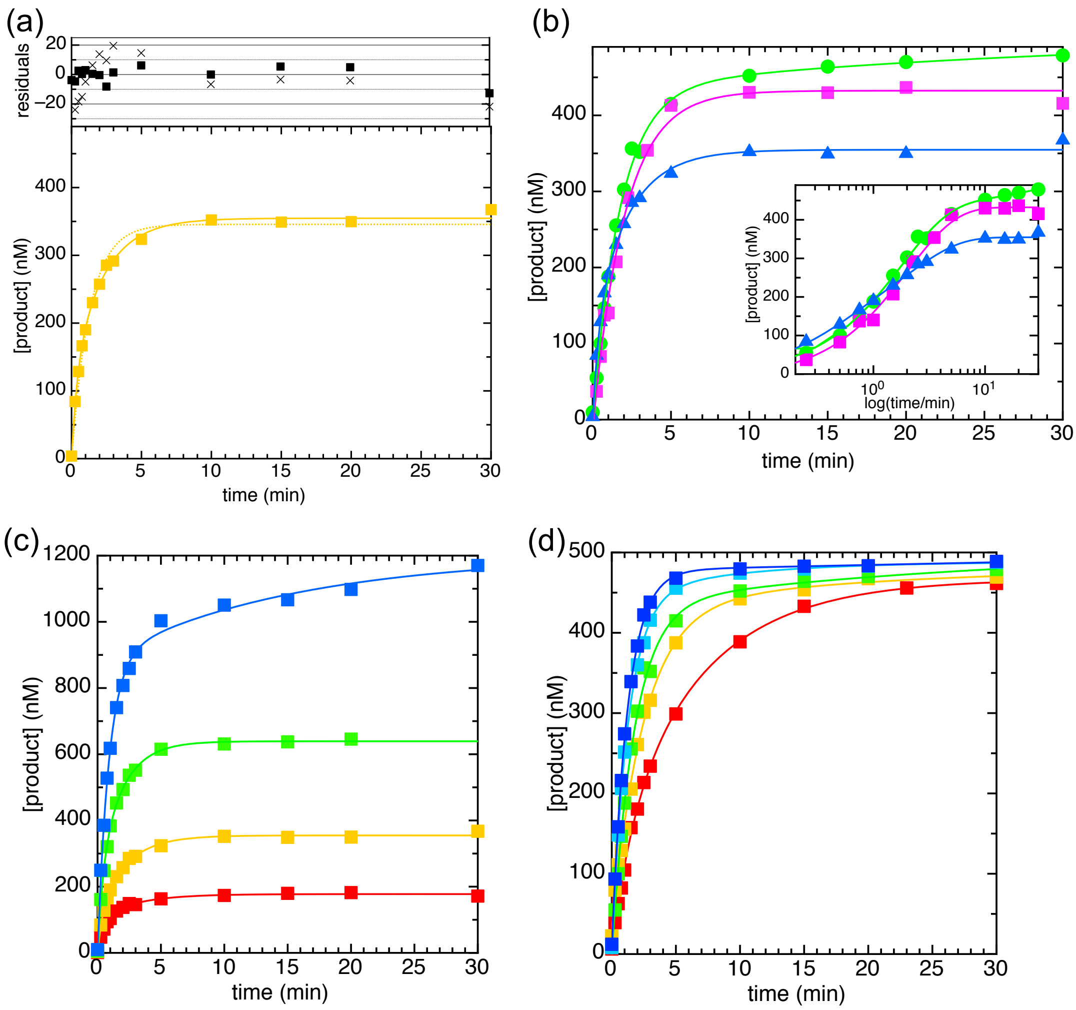

Fig. S4. Additional donor strand exchange assays. (a) Results of a DSE assay carried out at 20 ˚C using 500 nM FimD:FimC:FimH:FimG complex and 5 μM FimC:FimF$^{Q99C[A647]}$. The formation of the FimH:FimG:FimF$^{Q99C[A647]}$ product is plotted as a function of reaction time, with both a single exponential fit (dotted line; crosses in residual plot) and a double exponential fit (solid line; squares in residual plot). (b) Comparison of donor strand exchange assays after mixing: i) 500 nM FimD:FimC:FimH:FimG$^{S92C[A647]}$ with 5 μM FimC:FimF (green circles); ii) 500 nM FimD:FimC:FimH:FimG with 5 μM FimC:FimF (magenta squares; data from gel in Figure S3B, see Supplementary Methods for details of calculation); and iii) 500 nM FimD:FimC:FimH:FimG with 5 μM FimC:FimF$^{Q99C[A647]}$ (blue triangles). For clarity both real time (main figure) and log time (inset) are shown. The data sets with

labelled FimG or FimF fit best to double exponentials while the non-fluorescent data set appears to fit better to a single exponential. However, it should be borne in mind that densitometry from silver-stained gels is much less precise and sensitive than quantitation of fluorescence, so the second phase could be present but undetectable. What can be seen by comparison of the curves, however, is that the rates are very similar; this confirms that the presence of a label on position 92 of FimG or position 99 of FimF have little or no effect on the kinetics of donor strand exchange. (c) DSE assays as in panel A but with varying initial concentrations of FimD:FimC:FimH:FimG complex. The concentrations of FimD:FimC:FimH:FimG are: 250 nM (red), 500 μM (yellow), 1000 nM (green) and 2000 nM (blue). (d) Donor strand exchange assays performed at 20 ˚C between 500 nM FimD:FimC:FimH:FimG$^{S92C[A647]}$ and varying initial concentrations of FimC:FimF. Double exponential fits to the data are also shown. The concentrations of FimC:FimF are coloured by rainbow from lowest to highest and are: 1 μM (red), 2 μM (yellow), 5 μM (green), 10 μM (cyan) and 20 μM (blue). After fitting all five data sets to the model in Fig. 2a (see text for details), the best fit value for k_{DSE} is 2.1 ± 0.9 min^{-1}.

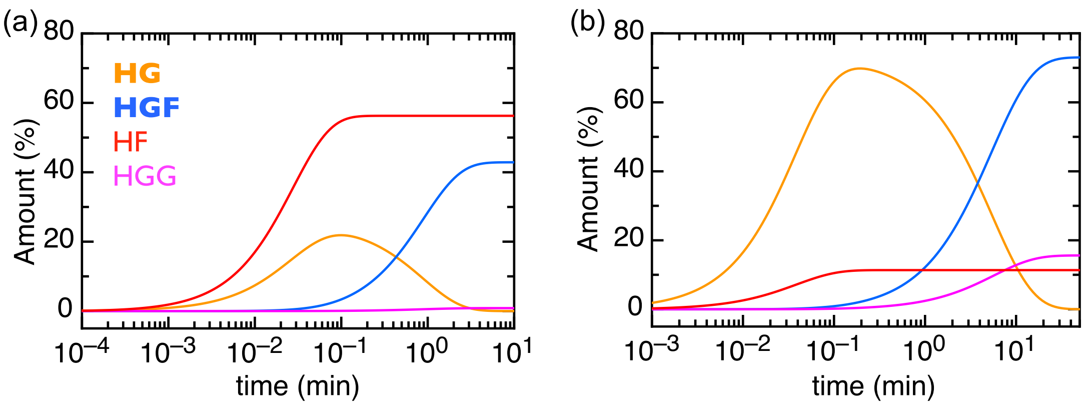

Fig. S5. Simulations of pilus tip formation. (a) Results of simulating the model in Fig. 4 with 100 nM FimD:FimC:FimH, 5 μM FimC:FimG and 5 μM FimC:FimF. Products are: FimD:FimC:FimH:FimG (orange), FimD:FimC:FimH:FimG:FimF (blue), FimD:FimC:FimH:FimF (red) and FimD:FimC:FimH:FimG:FimG (magenta). (b) The same as in panel A, but with only 0.5 μM FimC:FimF (10-fold less).

Supplementary References

1. Fersht, A. (W.H. Freeman & Company: 1985). *Enzyme Structure and Mechanism*.
2. Nishiyama, M. & Glockshuber, R. (2010). The outer membrane usher guarantees the formation of functional pili by selectively catalyzing donor-strand exchange between subunits that are adjacent in the mature pilus. *J. Mol. Biol.* **396**, 1–8
3. (2010). A capture coupling method for the covalent immobilization of hexahistidine tagged proteins for surface plasmon resonance. **627**, 91–100
4. van den Ent, F. & Löwe, J. (2006). RF cloning: a restriction-free method for inserting target genes into plasmids. *J. Biochem. Biophys. Methods* **67**, 67–74
5. Phan, G., Remaut, H., Wang, T., Allen, W.J., Pirker, K.F., Lebedev, A., Henderson, N.S., Geibel, S., Volkan, E., Yan, J., Kunze, M.B.A., Pinkner, J.S., Ford, B., Kay, C.W.M., Li, H., Hultgren, S.J., Thanassi, D.G. & Waksman, G. (2011). Crystal structure of the FimD usher bound to its cognate FimC-FimH substrate. *Nature* **474**, 49–53
